# Local Recurrence and Development of Spinal Cord Syndrome during Follow-Up after Surgical Treatment of Metastatic Spine Disease

**DOI:** 10.3390/cancers15194749

**Published:** 2023-09-27

**Authors:** Peter Knöll, Moritz Lenschow, Maximilian Lenz, Volker Neuschmelting, Niklas von Spreckelsen, Sergej Telentschak, Sebastian Olbrück, Maximilian Weber, Johannes Rosenbrock, Peer Eysel, Sebastian G. Walter

**Affiliations:** 1 Department of Orthopedics, Trauma Surgery and Plastic Surgery, Faculty of Medicine, University Hospital of Cologne, University of Cologne, Kerpener Str. 62, 50937 Cologne, Germany; peter.knoell@uk-koeln.de (P.K.);; 2 Department of General Neurosurgery, Center for Neurosurgery, Faculty of Medicine, University Hospital of Cologne, University of Cologne, Kerpener Str. 62, 50937 Cologne, Germany; moritz.lenschow@uk-koeln.de (M.L.); volker.neuschmelting@uk-koeln.de (V.N.); niklas.von-spreckelsen@uk-koeln.de (N.v.S.); sergej.telentschak@uk-koeln.de (S.T.); 3 Faculty of Medicine, University of Cologne, 50937 Cologne, Germany; 4 Department of Radiation Oncology, CyberKnife and Radiation Therapy, Faculty of Medicine, University Hospital of Cologne, University of Cologne, Kerpener Str. 62, 50937 Cologne, Germany; johannes.rosenbrock@uk-koeln.de

**Keywords:** ESCC, MSCC, spinal metastasis, decompression surgery, radiotherapy, spinal cord syndrome

## Abstract

**Simple Summary:**

Metastatic spinal disease is a devastating disease often associated with a reduced quality of life for patients. In these patients, quality of life can be reduced due to peripheral or axial pain, mechanical instability, fractures or neurological impairments. Treatment is interdisciplinary and, in many cases, includes surgery followed by radiotherapy. In some cases, patients develop novel neurologic deficits in the time after or during treatment. This study searches for risk factors leading to novel neurologic deficits. This study found a long time of more than 35 d between surgery and radiotherapy to be an important risk factor for occurrence of novel neurologic deficits.

**Abstract:**

Background: Surgical decompression (SD), with or without posterior stabilization followed by radiotherapy, is an established treatment for patients with metastatic spinal disease with epidural spinal cord compression (ESCC). This study aims to identify risk factors for occurrence of neurological compromise resulting from local recurrence. Methods: All patients who received surgical treatment for metastatic spinal disease at our center between 2011 and 2022 were included in this study. Cases were evaluated for tumor entity, surgical technique for decompression (decompression, hemilaminectomy, laminectomy, corpectomy) neurological deficits, grade of ESCC, time interval to radiotherapy, and perioperative complications. Results: A total of 747 patients were included in the final analysis, with a follow-up of 296.8 days (95% CI (263.5, 330.1)). During the follow-up period, 7.5% of the patients developed spinal cord/cauda syndrome (SCS). Multivariate analysis revealed prolonged time (>35 d) to radiation therapy as a solitary risk factor (*p* < 0.001) for occurrence of SCS during follow-up. Conclusion: Surgical treatment of spinal metastatic disease improves patients’ quality of life and Frankel grade, but radiation therapy needs to be scheduled within a time frame of a few weeks in order to reduce the risk of tumor-induced neurological compromise.

## 1. Introduction

Spinal metastases are a frequent diagnosis in about 15% of patients treated for an oncologic disease, and most likely this incidence is systematically underestimated [1,2,3]. In general and most cases, progression of spinal metastases is asymptomatic until the terminal phase. Up to one fifth of patients with spinal metastasis, however, experience the destruction of supporting spinal elements and develop symptomatic spinal cord compression [4]. Thus, symptomatic metastases to the spine, primarily and most often originating from malignancies in the breast, lung, prostate, and kidney, cause significant morbidity and reduced quality of life for those affected.

The spine’s structural complexity, crucial role in weight-bearing, and proximity to neural elements make it a critical site for metastatic infiltration, resulting in vertebral compression fractures, spinal cord compression, and neurological deficits. Amid the therapeutic options available, decompressive surgery combined with posterior stabilization emerges as an important intervention for managing the intricate interplay of mechanical instability and neural compromise that is inherent in metastatic spine disease. Other surgical treatment options are kyphoplasty (e.g., in combination with radiofrequency ablation or intraoperative radiotherapy—IORT), separation surgery, and minimal invasive techniques such as MIS or MASS [5,6,7,8].

Thus, surgical treatment is a valid option to ensure an improved quality of life in cases where mechanical instability or spinal cord compression is imminent or has already occurred [9,10]. In most cases where surgery is performed, reducing axial pain leads to an enhanced quality of life [11]. However, surgical treatment is only part of an interdisciplinary treatment including (neo-)adjuvant radiation as well as systemic therapy. Indication for surgery should always consider the overall health and performance status of the specific patient [9]. In cases of spinal metastases without impending mechanical instability or spinal cord compression, solitary radiation therapy may be a sufficient treatment as well [12]. However, tumor entity and the general health status of the patient are further important factors to consider when deciding on the best treatment [13]. Noteworthy, it is apparent that surgery cannot stand alone as a solution for metastatic spine disease when the demand for surgical techniques to improve biomechanical stability and neurological function is considered. Although interdisciplinary treatment regimens may improve quality of life, there are significant complications including the development of novel neurological deficits. This retrospective study of a large single-center cohort aimed to determine the risk factors for occurrence of novel neurological deficits and local tumor recurrence.

## 2. Materials and Methods

All consecutive patients who underwent surgery due to spinal metastases at our interdisciplinary spine center between January 2012 and March 2022 were assessed for inclusion in this study. The relevant data were collected from the center’s electronic database, which included medical records and radiologic images. Approval for the study was granted by the local ethics committee (approval code: 20-1643).

The following patient-related parameters were recorded: Age, gender, primary tumor histology, location within the spine, and medical comorbidities such as diabetes mellitus, coronary heart disease, history of smoking and chronic obstructive pulmonary disease (COPD), history of deep vein thrombosis, obesity (defined as a body mass index > 30), and osteoporosis. Multiple myeloma and lymphoma were summarized as hematopoietic cancers. Spinal instability was assessed using the Spinal Instability Neoplastic Score (SINS) and classified into stable (SINS 0 to 6), intermediate (SINS 7 to 12), and unstable (SINS 13 to 18) groups accordingly [14].

Surgical techniques such as decompression (laminotomy), hemi-laminectomy, laminectomy, and corpectomy were assessed. In cases of revision surgery due to local recurrence and novel neurological deficits, “debulking surgery” was registered as well. Additional dorsal instrumentation, kyphoplasty, or radiofrequency ablation were registered, too. Radiation therapy was executed by our department of radiation oncology using a standardized protocol. Here, the spinal tumor is irradiated with 30 Gy. This dose was applied hypofractionated by doses of 3 Gy in the definition of conventional external beam radiation therapy (cEBRT) [15].

The time period between the initial surgery and the start of radiation therapy at the surgically treated area was monitored, as well as perioperative complications such as wound healing disorders, wound infections, material dislocation, implant failure, epidural hematoma, and so on. Furthermore, the study assessed the Frankel grade (A = complete impairment; B = incomplete, sensory but no motor function below neurological level; C = incomplete, motor function preserved but majority of key muscles muscle grade < 3; D = incomplete, motor function preserved and majority of key muscles muscle grade > 3; E = normal) as well as the appearance of spinal cord syndrome, both before and immediately after surgery and during follow-up (more than 10 days after surgery) [16]. Finally, the degree of spinal cord compression before surgery and during follow-up was assessed by MRI using the Epidural Spinal Cord Compression (ESCC) scale and divided into a low (ESCC 0 to 1) and a high (ESCC 2 to 3) cord compression group [17].

All the scores were collected independently by at least two investigators. In cases of initial nonagreement, consensus was reached through case-based discussions.

The main outcome measures for this study were occurrence of novel neurological deficits or symptoms (motoric, sensory, or vegetative dysfunction as well as pain) during the course of follow-up as well as local recurrence detected by MRI or CT imaging in symptomatic patients. Hence, subgroup analysis was performed for all patients with spinal cord/cauda syndrome (SCS) during follow-up and for those without (NSCS).

### Statistical Analysis

Descriptive statistics were used to analyze clinical characteristics. Categorical variables were compared by a Chi-Square and a Fisher’s exact test. Continuous variables were tested for normal distribution using the Kolmogorov–Smirnov test. The data are reported with confidence intervals (CI) or as mean ± standard deviation. Group means from normally distributed data were compared using a two-sided unpaired Student’s *t*-test. A Mann–Whitney U test was used in the case of nonnormal distribution of data. All calculations were performed using SPSS software (Version 27, IBM SPSS Statistics for Windows, Armonk, NY, USA). A *p*-value < 0.05 was considered statistically significant.

## 3. Results

### 3.1. Demographics

In total, there were 810 patients who were assessed for final analysis. Of those, 64 had to be excluded due to incomplete records. The follow-up of all remaining 746 patients was 296.8 d (95% CI (263.5, 330.1)). The most common secondary diagnoses were COPD (23.4%) type II diabetes (19.6%) and atherosclerosis (15.8%). Overall survival one year after surgery was 62.2%.

### 3.2. Spinal Scores

**Figure 1:**
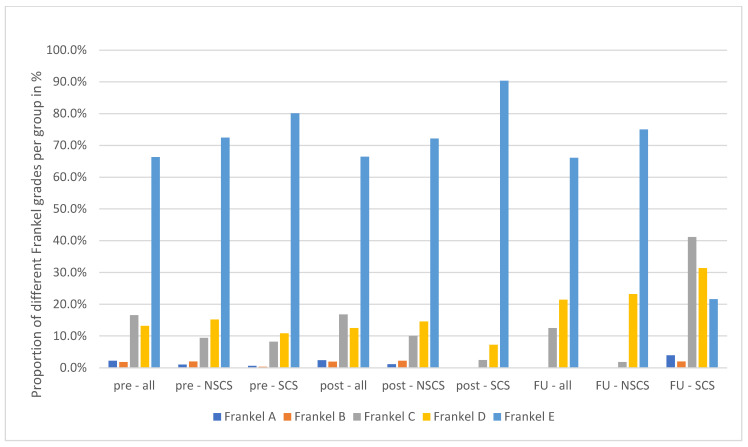
Shown is the distribution of pre- and postoperative Frankel grades among the whole cohort (all) and both subgroups with (SCS) and without (NSCS) spinal cord syndrome during follow-up (FU).

Initial ESCC was grade I (a–c) in 28.9% of cases; 36.1% of cases were grade II and 34.9% were grade III. There was no significant difference in distribution between patients that did not develop spinal cord syndrome (NSCS) during follow-up and those that did (SCS; *p* = 0.29; Table 1). The preoperative SINS score was intermediate on average (10.6 ± 4.9). The SINS score was not significantly different between the NSCS and the SCS subgroups (10.5 ± 3.1 respective 10.0 ± 2.8; *p* = 0.55). The Frankel grades are depicted in Figure 1.

### 3.3. Additional Treatment

In this cohort, 53.4% of the patients received adjuvant systemic therapy (chemotherapy: 42.1%—different regimes depending on tumor entity, targeted therapy: 11.3%—different regimes depending on tumor entity and molecular profile); 12.1% of the patients received preoperative embolization of the affected vertebral body. There was no significant difference between the NSCS and SCS subgroups (*p* = 0.73).

### 3.4. Complications

In total, 188 patients (25.2%) required revision or secondary surgery, and there was no significant difference between the NSCS and SCS subgroups (*p* = 0.67). 42.5% of these cases were stabilized ventrally through piecemeal vertebrectomy and implantation of a vertebral body implant via secondary surgery. 84.3% of revision surgeries were performed on the same vertebral segment. Deep wound infections (6.0%), implant loosening or breakage (8.9%), epidural hematoma (5.2%), dura injury (2.5%), and surgical intervention for superficial wound infections (epi-fascial; 14.3%) were further reasons for revision surgery, with no significant differences between both subgroups (*p* = 0.31).

Other perioperative complications were deep vein thrombosis (2.0%), pneumonia (3.1%), cardiac events (1.1%), and central nervous system events (0.7%).

## 4. Discussion

Improving quality of life for patients with metastatic spinal disease is the major goal of surgical treatment [18]. Most often, quality of life is enhanced through pain relief, gaining local tumor control respective to controlling metastatic disease at the treated site, improving neurological deficits, maintaining or ameliorating functional status, and preventing further mechanical instability. Surgically this is achieved by removal of the metastatic deposit, and prevention or correction of deformity with stabilization and/or decompressive neurolysis. The latter may be essential for the recovery of an impaired neurological status if a spinal cord syndrome has already occurred.

Patients undergoing this kind of treatment are highly vulnerable and at high risk of a plethora of medical problems. In fact, complication rates are reported to be as high as 47% [19,20,21,22,23]. Therefore, decision making on which patients to operate on is essential to ensure that patients benefit from surgery [24]. In all cases, the aims of the surgical intervention need to be defined, and a careful weighing of the invasiveness of surgery and specific risk factors in relation to the patient’s physiological condition and prognosis has to be performed.

In addition to immediate and perioperative complications, patients may develop a local recurrence that may become apparent with novel neurological compromise [25]. To the best of our knowledge, this is the largest study to investigate risk factors for the development of novel neurological compromise after surgical treatment for metastatic spine disease. In this retrospective study, 746 cases treated for metastatic spine disease over the course of more than 10 years were reviewed for development of a spinal cord syndrome without temporal correlation to previous surgical measures. Fifty-six (7.5%) cases developed a spinal cord syndrome during follow-up and allowed thus for a comparison with 690 (92.5%) cases that did not experience spinal cord syndrome during follow-up. Both subgroups were comparable in terms of demographic characteristics, and there were no significant differences in comorbidities, surgical techniques, initial KPS, ESCC, or SINS.

In this cohort, no significant difference in complication rates was observed between both subgroups. The complication rates were comparable to those previously reported in the literature [22,26].

The main endpoint of this study was the development of neurological compromise due to local recurrence. As parameter the Frankel score was used, revealing a significant difference (*p* = 0.02) between both subgroups with a deterioration in the SCS subgroup during follow-up, which was not the case immediately before respective to after surgery (Figure 1 and Table 2). Deteriorations in Frankel grade within the SCS subgroup were due to local recurrence objectified by novel CT or MRI scans. The main risk factor identified for development of a symptomatic local recurrence was delayed radiation therapy. In fact, time to radiation was significantly prolonged in the SCS group (*p* < 0.001), and it should be discussed whether this timeframe should not exceed five to six weeks. As complications and revision surgery rates were not significantly higher in the SCS group, prolonged time to radiation can be attributed to decentral patient management (e.g., patients who were treated surgically at our center and planned to receive radiation therapy externally) and lack of awareness on the part of healthcare providers of the need to schedule early radiation therapy. Interpreting our data and backed up by our experience in clinical routine, delay to radiation therapy is often multifactorial and cannot be associated with a particular error in patient management. It has to be stated that adjuvant radiation does not necessarily exclude the risk of local recurrence, but rates are lower than reported in this cohort [12,27,28]. These results once again emphasize the importance of a scheduled interdisciplinary treatment for achieving favorable midterm outcomes. For future treatment, it remains to be discussed whether the established protocol (decompression and instrumentation plus cEBRT) or separation surgery with or without PEEK instrumentation plus stereotactic radiation therapy will truly yield higher benefits for patients [29,30].

To our knowledge, no randomized controlled trials exist for this patient cohort to determine the optimal time for adjuvant radiation therapy. Possibly, neoadjuvant radiation therapy may not be inferior, but data are still scarce [31].

Besides the inherent limitations of a retrospective study, our results are from a single center. Nevertheless, the overall number of cases included is high.

## 5. Conclusions

Surgical treatment of spinal metastatic disease provides significant benefits to patients’ quality of life, but radiation therapy must be scheduled within a few weeks in order to reduce the risk of tumor-induced neurological compromise.

## Figures and Tables

**Table 1:** Data on all patients included in this study and of both subgroups—those with spinal cord syndrome during follow-up and those without. Note that Frankel grade distribution is provided in %. *p*-Values marked with an * were significant (<0.05).

	Total	No Spinal Cord Syndrome during Follow-Up	Spinal Cord Syndrome during Follow-Up	*p*-Value
Number of cases n (%)	746	690 (92.5%)	56 (7.5%)	
Tumor entity n (%) 1 = Renal Ca 2 = Mamma Ca 3 = Lymphoma 4 = SCLC 5 = NSCLC 6 = Thyroid Ca 7 = Multiple Myeloma 8 = Prostate Ca 9 = Sarcoma 10 = CUP 11 = GI 12 = Urothel 13 = Malign. Melanoma 14 = other	45 (6.0%) 116 (15.5%) 35 (4.7%) 17 (2.3%) 115 (15.4%) 17 (2.3%) 71 (9.5%) 117 (15.7%) 22 (2.9%) 35 (4.7%) 73 (9.8%) 21 (2.8%) 17 (2.3%) 45 (6.0%)	41 (5.9%) 111 (16.1%) 33 (4.8%) 15 (2.2%) 106 (15.3%) 15 (2.2%) 63 (9.1%) 106 (15.3%) 21 (3.0%) 33 (4.8%) 70 (10.1%) 19 (2.7%) 15 (2.2%) 41 (5.9%)	4 (7.1%) 5 (8.9%) 2 (3.6%) 2 (3.6%) 9 (10.1%) 2 (3.6%) 8 (14.2%) 11 (19.6%) 1 (1.8%) 2 (3.6%) 3 (5.4%) 1 (1.8%) 2 (3.6%) 4 (7.1%)	0.56
Gender (M/F)	463/283	423/267	39/17	0.31
Age (in yrs.)	63.7 (50.5, 77.0)	64.0 (51.3, 76.8)	60.6 (51.8, 74.2)	0.54
t to radiation (d; 95%CI)	31.8 (12.3, 51.3)	22.6 (10.6, 34.7)	106.2 (44.9, 167.1)	* < 0.001
KPS	61.0 ± 14.4	61.0 ± 14.4	62.5 ± 13.4	0.44
Frankel grade presurgery (in %) A/B/C/D/E	2/2/17/13/66	2/2/17/13/66	0/0/13/21/66	0.47
Frankel grade post-surgery (in %) A/B/C/D/E	1/2/9/15/72	1/2/10/15/72	0/0/2/23/75	0.16
Frankel grade follow-up (in %) A/B/C/D/E	1/1/8/11/79	0/0/2/7/90	4/2/41/31/22	* 0.02
Tumor Main Location Cervical Thoracic Lumbar Sacral	11.8% 53.9% 32.6% 1.9%	12.8% 56.1% 29.3% 1.8%	16.1% 53.6% 25.0% 5.4%	0.76
Type of surgery Decompression surgery (DS) Hemilaminectomy Laminectomy No decompressive surgery Vertebrectomy Debulking surgery	3.4% 7.2% 68.7% 10.3% 9.4% 1.1%	3.4% 7.3% 68.7% 10.2% 9.4% 1.0%	3.6% 5.4% 67.9% 10.7% 10.7% 1.8%	0.82

**Table 2:** Overview on previous history of a symptomatic spinal cord syndrome (SCS) in patients who developed such symptoms during the course of follow-up. Note that inapparency of SCS before initial surgery is not predictive of a better outcome.

	Spinal Cord Syndrome during Follow-Up
Spinal cord syndrome without prior SCS	40 (76.9%)
Spinal cord syndrome with prior history of SCS	7 (13.5%)
Aggravated SCS	9 (17.3%)
ESCC score (I/II/III in %)	25.2/39.1/35.7

## Data Availability

The datasets generated and/or analyzed in this study are available upon reasonable request.
